# BME 2.0: Engineering the Future of Medicine

**DOI:** 10.34133/bmef.0001

**Published:** 2023-01-25

**Authors:** Michael I. Miller, Andrew O. Brightman, Frederick H. Epstein, K. Jane Grande-Allen, Jordan J. Green, Eileen Haase, Cato T. Laurencin, Elizabeth Logsdon, Feilim Mac Gabhann, Brenda Ogle, Chun Wang, George R. Wodicka, Rai Winslow

**Affiliations:** ^1^ Department of Biomedical Engineering, Johns Hopkins University, Baltimore, MD, USA.; ^2^ Weldon School of Biomedical Engineering, Purdue University, West Lafayette, IN, USA.; ^3^ Biomedical Engineering, University of Virginia, Charlottesville, VA, USA.; ^4^ Department of Bioengineering, Rice University, Houston, TX, USA.; ^5^ Department of Biomolecular Engineering and Department of Orthopaedic Surgery, University of Connecticutt, Storrs, CT, USA.; ^6^ Department of Biomedical Engineering, University of Minnesota-Twin Cities, Minneapolis, MN, USA.

## Abstract

If the 20th century was the age of mapping and controlling the external world, the 21st century is the biomedical age of mapping and controlling the biological internal world. The biomedical age is bringing new technological breakthroughs for sensing and controlling human biomolecules, cells, tissues, and organs, which underpin new frontiers in the biomedical discovery, data, biomanufacturing, and translational sciences. This article reviews what we believe will be the next wave of biomedical engineering (BME) education in support of the biomedical age, what we have termed BME 2.0. BME 2.0 was announced on October 12 2017 at BMES 49 (https://www.bme.jhu.edu/news-events/news/miller-opens-2017-bmes-annual-meeting-with-vision-for-new-bme-era/). We present several principles upon which we believe the BME 2.0 curriculum should be constructed, and from these principles, we describe what view as the foundations that form the next generations of curricula in support of the BME enterprise. The core principles of BME 2.0 education are (a) educate students bilingually, from day 1, in the languages of modern molecular biology and the analytical modeling of complex biological systems; (b) prepare every student to be a biomedical data scientist; (c) build a unique BME community for discovery and innovation via a vertically integrated and convergent learning environment spanning the university and hospital systems; (d) champion an educational culture of inclusive excellence; and (e) codify in the curriculum ongoing discoveries at the frontiers of the discipline, thus ensuring BME 2.0 as a launchpad for training the future leaders of the biotechnology marketplaces. We envision that the BME 2.0 education is the path for providing every student with the training to lead in this new era of engineering the future of medicine in the 21st century.

## Introduction

The last 70 years has seen the rapid growth in our ability to acquire, store, and analyze information about the external world. This has been enabled by an accelerating trajectory of digital tools: from the first computers, through read/writable storage, to search engines that launched the information age of big data and the ubiquitous sensors of the internet of things. Scientific progress has rewritten the rules for exploration and control of the external world, enabling exploration of space and the deepest oceans; optimization of energy grids and air traffic networks; taller, greener cities; and a navigator on our phones. The external world around us has been measured, analyzed, and changed through engineering.

The greatest opportunities for disruptive change during the 21st century are likely to be in the measurement, analysis, control, and augmentation of the internal world of the human body. Rapid advances are being made possible by a new accelerating trajectory of enabling tools based on advances in imaging, genomics, systems medicine, molecular and cellular engineering, and other biomedical sciences. As with digital storage, we can now not only “read” genes but also “write” genes. Just as every position on the globe has global positioning system (GPS) coordinates, the human body is also being mapped, cell by cell. The National Institutes of Mental Health Brain Initiative Cell Census Network (BICCN) project is cataloguing every cell type in the most complex structure in the known universe—the brain [1]; the Intelligence Advanced Research Projects Activity (IARPA) Machine Intelligence from Cortical Networks project is mapping the brain, neuron by neuron [2]. The possibilities that arise as engineering principles as applied to human health are endless and life changing [3]. The genetic revolution is coming [4].

At the same time, just as earlier industrial revolutions have powered new generations of engineering technologies for understanding the external world, the new third industrial revolution [5] enabled by digital manufacturing and 3-dimensional printing is ushering in a new age of engineering technologies for the biomedical world. Our new generation of engineering innovators will be manufacturing complex, personalized human tissues and organs for transplant and augmentation. From writing genes to building new organs, the field of biomedical engineering (BME) is and will be at the forefront of these accelerating disciplines. To match the pace of change in tools and applications, we need an educational curriculum that prepares students for applying cutting-edge tools that are already exploding the frontiers of measurement at every scale of the human, from single-molecule measurements to wearable wireless devices, as well as the remarkable advances in the computational sciences and machine learning and artificial intelligence [6]. As we enter what many are calling the fourth industrial revolution of artificial intelligence and machine learning [7], BME will be a biomedical data-driven discipline with our trainees forming the next generations of biomedical data scientists [8].

The undergraduate curriculum that has served us well for the first 50 years of BME must evolve to support new innovation. This would not be the first time a classic undergraduate curriculum has undergone a necessary redesign. Physicists had to balance the classical training of 19th century physics with the explosive developments of quantum physics, and in so doing enabled the interdisciplinary sciences that led to semiconductors and solid-state electronics. We too are at a similar inflection point in undergraduate BME education, where the acceleration of new tools and techniques enables interdisciplinary insights, this time into human health and therapies. Thinking historically with the emergence of graduate programs, BME as a discipline emerged in the 1960s and 1970s, birthed early by several cofounders at Case Western University, Duke University, Johns Hopkins University, University of Minnesota (UMN), University of California San Diego, University of Pennsylvania, and University of Rochester programs, several of the programs founded in the 1960s established as divisions within schools of medicine with undergraduate BME curriculum beginning in the 1970s. Herman Schwann and Sam Talbot were early founders at Penn and Johns Hopkins University, respectively (https://ethw.org/Oral-History:Richard_J._Johns). The curricula were designed as an outgrowth of the more mature engineering disciplines such as electrical engineering (EE), mechanical engineering (ME), and materials science (MS) or chemical engineering (ChE) pathways. Students would declare an area of concentration in EE, ME, MS, or ChE; those students interested in studying neurons or the pacing of the heart would choose focus area concentrations in EE and those interested in biomaterials MS or ChE. As the field has matured, the interdisciplinary nature of applying engineering principles to biology and medicine has became central. Studying the heart or building an auditory or visual neuroprosthesis does not afford BMEs the luxury of studying any single systems theory methodology in isolation, but rather must engage the myriad of chemo–electro–mechano systems.

## BME 1.0 and the Whitaker Foundation Era

During the 1980s, BME departments were relatively limited. That changed during the transformational era of the Whitaker Foundation years of 1991 to 2006. The Whitaker Foundation was established in 1976, and in 1991, it decided that it would heavily invest all of its resources to enhance biomedical engineering education, that ending in 2006. Spending increased dramatically during this period as a result of this decision, during which period Whitaker invested all of its resources on enhancing BME education resulting in the creation of many new BME undergraduate programs around the country [9], many of which were exclusively within schools of engineering. Well over a hundred programs exist today. This impact was transformational, and it is likely that the Whitaker Foundation’s influence on the growth of BME as a formal Engineering discipline ultimately gave rise to the establishment on December 29 2000 of National Institute of Biomedical Imaging and Bioengineering (NIBIB) focused on biomedical and biological engineering. NIBIB was signed into law by the then President William Jefferson Clinton on December 29, 2000 via the National Institute of Biomedical Imaging and Bioengineering Establishment Act (H.R. 1795) (https://www.nih.gov/about-nih/what-we-do/nih-almanac/national-institute-biomedical-imaging-bioengineering-nibib).

It was during this period that both Johns Hopkins University led by Murray Sachs and the University of California at San Diego led by Shu Chien received the first Whitaker Foundation Leadership Awards in the country. In 1998 [10], the Whitaker Foundation’s investment was the crucial beginning of many such awards for transforming BME across the country, bringing with it a wave of new energy. These awards required that the new BME departments be built within the schools of engineering and, equally important for this treatise, required the development of a robust BME undergraduate curriculum.

The Whitaker Foundation appreciated the importance of growing the discipline of BME and formalizing curriculum development for the field to flourish. The Whitaker award enabled the hiring and support of engineers conducting BME research, including grants to 1,500 junior faculty, 400 BME graduate student fellowships, and 110 one million dollar awards to hire BME faculty [9]. It was during this period that the earliest BME specific curricula were being enhanced and developed, what we call in this report “BME 1.0,” focusing on training multilingual biomedical engineers, trained in both biomedicine and computational and analytical modeling. The BME 1.0 curriculum was an integration of engineering principles taught from the perspective of biomedicine. Students in BME 1.0 were beginning to identify as engineers trained in this new discipline of engineering medicine and biology, and would no longer identify as belonging to one traditional engineering discipline or the other.

The time was right for change, and BME would not remain the same field as we turned the corner of the 21st century. Quoting Paul Citron and Robert Nerem in 2004 [11]:

[originally] biomedical engineering was largely involved in the application of more traditional engineering disciplines – e.g. chemical, electrical, and mechanical engineering – to problems in medicine and biology (Paul Citron)

what [has] emerged is a new engineering discipline, arguably the only new one in the twentieth century, an engineering discipline that is biology-based (Robert Nerem)

We recently celebrated the 50th anniversary of the Biomedical Engineering Society (https://www.bmes.org/bmes-50-years), an ideal time to consider the philosophy and principles that underlie our next generation of BME education. The next 50 years of understanding biology requires integrative thinking about complex dynamical biologic systems that are heterogeneous in space and changing in time. At the same time, the economics of biomedical technologies have led to a democratization of biosciences so that subjects that might have once focused on theory alone due to the paucity of data, now because of the ubiquity of biosensors—both physical and molecular—provide students with the opportunity to conduct their own research and design projects. In introductory biomedical data science classes, we can use public datasets as motivating examples and case studies, including public health datasets like the National Health and Nutrition Examination Survey (https://www.cdc.gov/nchs/nhanes/index.htm/) and medical imaging and MRICloud [12], public data and tools (https://www.nitrc.org/projects/mricloud/) and open EEG (https://github.com/meagmohit/EEG-Datasets/) and biosignal data (https://www.hindawi.com/journals/cin/2011/935364/), and open access educational AI/ML benchmark datasets like medical MNIST (https://medmnist.com/) as well as Intensive Care MIMIC (https://www.nature.com/articles/sdata201635) and the Philips eICU (https://www.nature.com/articles/sdata2018178/) databases. The Open Case studies project (https://www.opencasestudies.org/) is an invaluable resource for data, such as the public opioid database creating the opportunity to teach lessons in data wrangling, merging, and exploratory graphics. More than ever, the community can be engaged in building a new generation of data-driven biomedical engineers.

## BME 2.0: A Roadmap of Disciplines for Next-Generation BME Education

We propose the next generation of integration of engineering disciplines in BME, what we term “BME 2.0,” which builds on the process that was initiated during the early post-Whitaker years. BME 2.0 was first announced at the BMES 50th celebration (https://www.bme.jhu.edu/news-events/news/miller-opens-2017-bmes-annual-meeting-with-vision-for-new-bme-era/). BME 2.0 will be enabled by tight coupling with the biomedical enterprise within schools of medicine as well as the biotechnology, pharamaceutical and medical technology industries. We envision a curriculum with focus areas that builds on the major discoveries and emerging opportunities in biomedical research. To deal with the dynamism of the biomedical science discovery process, a principle for evolution of the BME 2.0 curriculum is that the most important research discoveries are rapidly conserved through their integration into the BME 2.0 curriculum.

A substantial departure from the BME 1.0 transition is motivated by the remarkable progress that we have seen in the biomedical sciences at the molecular and cellular scales. The frontiers of BME including molecular, genetic, and microphysiological systems are being tightly integrated into the BME programs, bringing together the natural sciences core, biology–chemistry–physics, in new fundamental ways. The BME 2.0 curriculum would begin for the entering class with an in-depth introduction to modern life sciences and the scientific foundations of modern molecular and cell biology, followed by a second year of rigorous quantitative bioengineering foundations. In junior and senior years, students would explore their areas of interest in their practice of the discipline differentiating into focus areas.

**Fig. 1.**
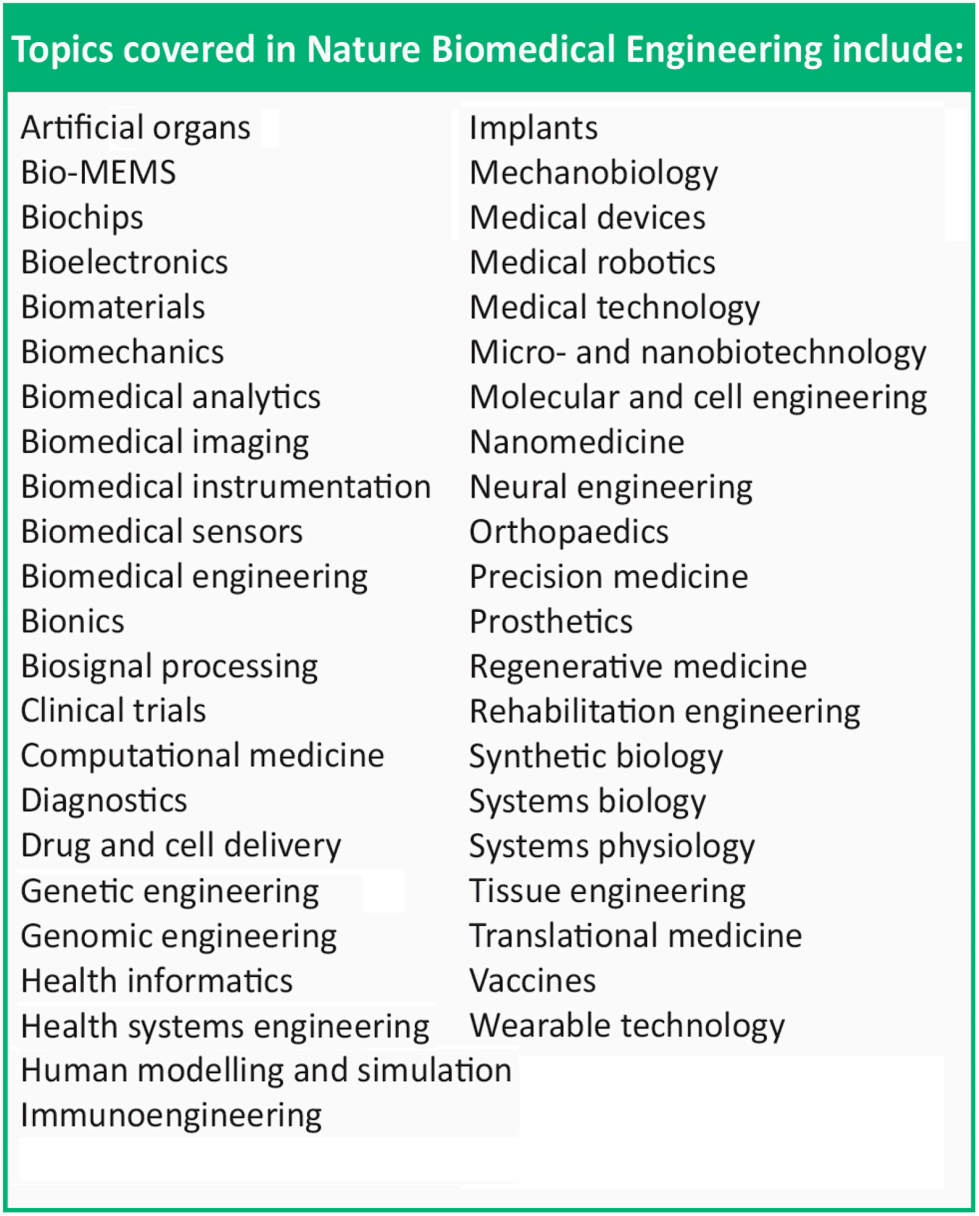
Topics in nature biomedical engineering [42] largely represent the BME focus areas, which are emerging nationwide including biomedical imaging and medical devices, biomechanics and mechanobiology, computational medicine, genetic engineering and systems biology, digital health and biomedical data science, neuroengineering, and immuno, regenerative, and tissue engineering.

The accompanying Fig. 1 shows the table of specialty areas of BME published by *Nature Biomedical Engineering*, which instantiate in part the focus areas that have emerged in many of the schools nationwide, including biomedical imaging and medical devices, biomechanics and mechanobiology, computational medicine, genetic engineering and systems biology, digital health and biomedical data science, neuroengineering, and immuno, regenerative, and tissue engineering.

Biomedical engineers will be trained in biomedical data science, which intersects all of the focus areas. The BME 2.0 curriculum ideally would provide students with the flexibility in their junior and senior years to fully personalize their education according to interests and major focus areas. While having flexible junior and senior years that enable student exploration in focus areas, design projects, and independent research projects is a critical component of some BME programs today, it is still not the standard in programs today.

BME 2.0 also envisions a new type of engagement in which the educational programs of our BME students recognize insights garnered from clinical and industrial partnerships that articulate the need for new therapeutics, diagnostics, and biomedical enabling technologies. Examples that are already emerging from this include design programs that integrate clinical faculty including BS/MD programs, PhD/MD and MD/MS programs, as well several engineering-based medicine programs including the Carle Illinois College of Medicine as one of the first medical schools with an engineering-based curriculum, with EnMed at Texas A&M College of Medicine and Duke and Minnesota all having MD-MSE dual degree programs. Vanderbilt has a Medical Innovators Development Program focused on training physicians in innovation/design for which the MDs must have existing engineering degrees to participate. Industrial involvement in BME curriculum is progressing from a distant, advisory relationship to synergistic resources in which universities in regions with high concentrations of medical technology industries are engaged in partnering models that provide opportunities for student project translation as a real-world educational experience, a well known example the Coulter Foundation.

We believe that this next generation of differentiated curriculum responds directly to the question, “Why should we hire biomedical engineers rather than chemical, electrical, or mechanical engineers?”. The BME answer is: Biomedical engineers are already being trained in the converging technologies of the biomedical marketplace. They are bilingually fluent in biology and mathematically based model thinking, working well in teams within highly regulated translational environment.

## BME 2.0: Engineers from Day 1

Clearly, with the creation of biological and bioengineering tools accelerating, it is the right moment to chart a new course in what and how we teach. As discoveries and inventions move faster and faster, the democratization of advanced scientific tools implies that our students require earlier access. We believe that the BME 2.0 model for undergraduate education presents the opportunity for a new kind of vertical integration [13], defined by a diverse and multigenerational learning environment for freshmen–sophomores–juniors–seniors, learning, doing, and teaching. Students would not just be learning but would be practicing the discipline: sequencing genomes, engineering stem cells, designing synthetic human chromosomes, building imagers, engineering improved healthcare approaches using big data, and other real-world projects with basic science and clinical impact.

In support of this notion, we see that with nationwide advances in STEM (science, technology, engineering, and mathematics) education at the high school level, today many of our college students are better prepared in key subject matter, including math and biology, and they are eager for advanced undergraduate training. Many of our students are doing activities to prepare them for more advanced training including attending immersive summer programs, and because of early interest, many are attending STEM high school with Advanced Placement (AP) courses, with the national average increasing from 20% to 33% between 2004 and 2014 [14,15]. States including MD, VT, MA, CA, NJ, CT, VA, and NY have high rates of students getting at least a 3 in their AP math exams [16]. A key guidepost for BME 2.0 should therefore be that our students have the opportunity to be practicing biomedical engineers from day 1, not waiting until their senior year in college or post-graduation before they do BME work. To this end, we must translate the greatest aspects of the biomedical discovery enterprise throughout the undergraduate curriculum.

This philosophy meshes well with leading educational practice; the students benefit enormously from a curriculum that emphasizes the more advanced components of active learning—creation of new devices, data, products, or knowledge and self-evaluation and analysis of a student’s own projects—and provides the mentoring and leadership opportunities that will enable students to succeed both pre- and post-graduation. To accomplish this, BME 2.0 is built around several principles.

### Educate students bilingually, from day 1, in the languages of modern molecular biology and the analytical modeling of complex biological systems

Traditionally, undergraduate engineering programs begin with a baseline year of math, physics, and chemistry courses upon which the remaining curriculum is built. This implies that students are delayed from engaging with BME-specific learning that enables them to identify their interests and to understand the context of their other coursework. We are advocating an increase of engagement of students in their freshman year that includes a life sciences foundations course in which students study molecules, cells, tissues, organs, and diseases, emphasizing the quantitative nature of biology and the biochemistry of biological structures, exploring the multiscale nature of tissues and the dynamics of multicellular morphogenesis. This continues a trend in BME programs requiring freshman engineering curriculum lead by BME faculty [17]. For the sophomore year, we propose a quantitative BME boot camp, teaching analytical principles of biological modeling and simulation with the material designed to prepare students for representing and modeling both linear and nonlinear systems that recur throughout the BME undergraduate curriculum. From the life sciences and quantitative BME foundations, students would emerge as quantitatively trained engineers that own biological and medical thinking and problem formulation. They would be fluent in the math and physics of quantitative systems analysis, control of complex dynamical systems, and molecular and cellular biology and physiology.

**Fig. 2.**
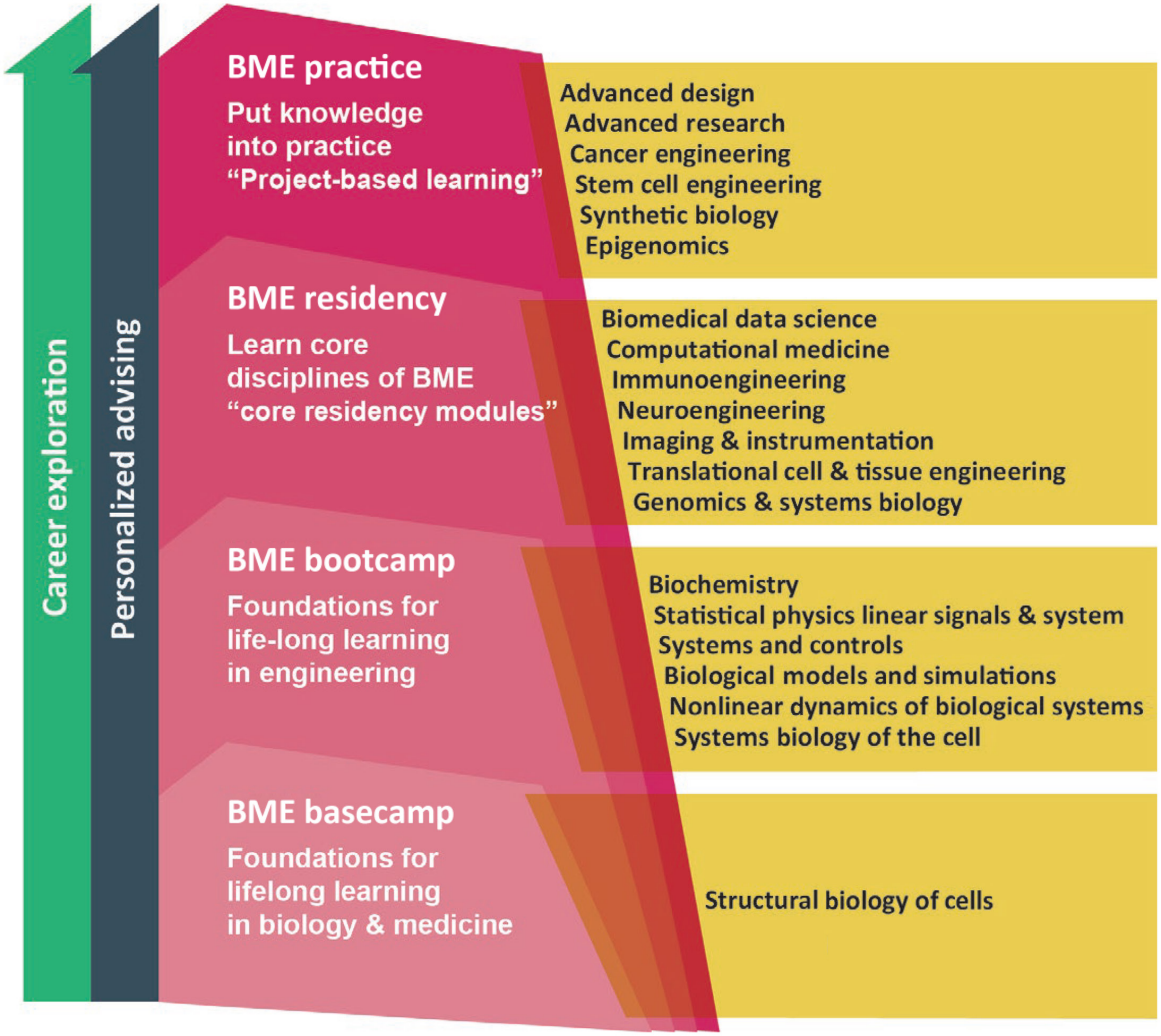
Representation of the modern BME 2.0 curriculum shown following a vertically integrating pyramid structure in which all activities continue throughout the 4 years: 2 years of advanced BME practice built upon a foundation of life science and quantitative training. All 4 years feature project-based learning coupled with translation and/or clinical insertion as an endpoint.

The third and fourth years should bring the student to hands-on and project-focused coursework, which reflect their differentiating interests and goals and prepare the students to practice within the field through project-based laboratories and classes oriented to their future careers in medical devices, biotechnologies, and healthcare analytics. This is depicted in Fig. 2.

Interestingly, we believe these 2 years of BME 2.0, life science foundations and quantitative BME boot camp, are well encapsulated by the Harvard Medical School admission requirements:

because biology is the most elegant expression of chemistry, physics, and mathematics, computational skills that tie these previously separate disciplines together should be emphasized [18].

The first 2 years of BME 2.0 builds a solid foundation in courses lead by BME faculty focused on BME applications. BME 1.0 required students to focus in mechanical, electrical, or materials engineering and take courses from other departments in signals and systems, thermodynamics, and mechanics. BME 2.0 has foundational courses that include biomechanics, bioelectricity, and biomaterials. Once students have learned how engineering principles apply to physiological, cellular, and genomic systems, they can design their own advanced courses, such as an advanced focus area class such as tissue engineering or biomedical data science, and or an advanced design team or research project allowing students to gain expertise far beyond the classroom [19–22].

### Prepare every student to be a biomedical data scientist

In the junior year, every student will have had statistics and linear algebra and are therefore prepared to take biomedical data science. We believe that biomedical data science training is critical for all students in BME—no matter where their professional goals lie, from genetic systems to regenerative engineering to careers in medicine. As discussed by the Council of Chairs of Bioengineering and Biomedical Engineering, there is a growing consensus among BME programs that data science education that teaches not only how to model and interpret complex data but also how to wrangle data is of critical value to BME as a discipline [23]. An undergraduate biomedical data science curriculum for BME will establish core skills in programming and data wrangling for varied applications.

**Fig. 3.**
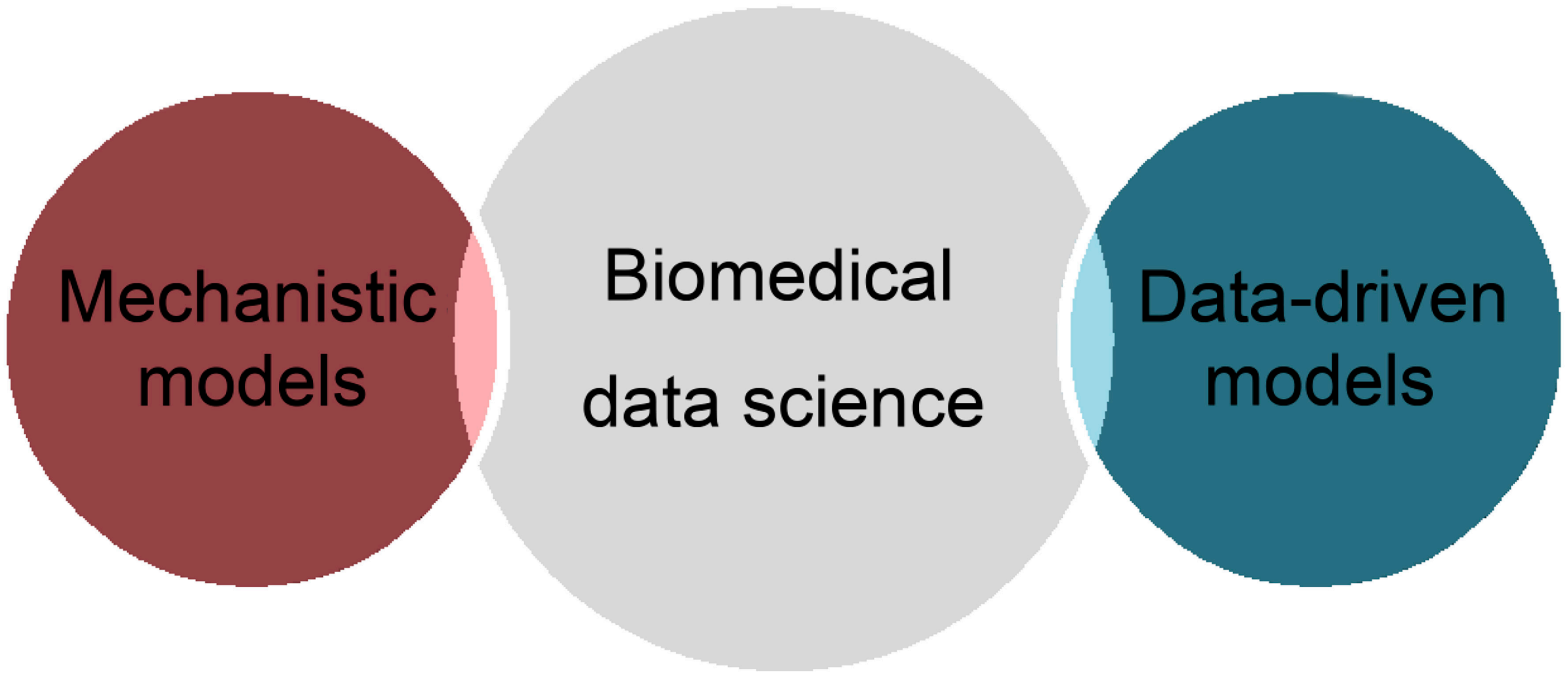
Depicting biomedical data science as a marriage between classical systems and computational mechanistic modeling of complex biological systems coupled to the data-driven discovery modeling of modern machine learning. These 2 modeling approaches together form the effective pairing of biophysics-informed modeling.

Biomedical data science uniquely bridges our more classical approaches of systems and analytical approaches to mechanistic modeling of biomedical systems, with the modern approaches of data-driven discovery. With the recent emergence and emphasis on incorporating biophysical models into data science via physics-informed machine learning [24], it is clear that understanding the evolutionarily complex biological systems central to BME by incorporating mechanistic models is an essential part of biomedical data science. With the lowering of barriers for data collection, biologically inspired mechanistic models coupled to data-driven model discovery form the central lynchpins of modern biomedical data science. This is depicted in Fig. 3.

Students will complement their systems modeling techniques by learning data science methods including statistical inference, dimensionality reduction techniques, unsupervised and supervised machine learning, and graph-based techniques and use of cloud computing resources. Examples can be drawn from data analysis of cutting-edge techniques to analyze genomics, transcriptomics, metabolomics, proteomics, physiological, and imaging data to gain a deeper appreciation for how to frame and test biological hypotheses from molecular to tissue scales. Students will learn to choose modeling techniques appropriate to addressing biological problems at different scales, quantitative characterization of biological properties, parameter estimation and sensitivity analysis, model verification and validation, and integration of computational modeling with experimental approaches.

### Build a unique BME community for discovery and innovation via a vertically integrated and convergent learning environment spanning the university and hospital systems

BME is of the fastest progressing disciplines of discovery and innovation in biology, biomedicine, and biotechnology. Building discovery and innovation into the curriculum with projects that incorporate laboratory-based research experiences is essential. Having research and team-based projects as part of the cutting-edge laboratories has many benefits for developing skills that are essential to all career paths.

Bringing students together in interdisciplinary and multiclass project teams was introduced early on by Rice et al. [13], with the students working in teams on projects both in the classroom and in their research experiences. There are many examples of universities implementing the student–teaching–student model in which problem solving engages undergraduates in multigenerational teams engaging the full ecosystem of graduate students, clinicians, and practicing engineers [25]. The full spectrum of BME design, from clinical needs assessment and prototyping to translation into clinical solutions, is a central element of the BME 2.0 vertically integrated curriculum. Empowering students to apply for external research opportunities also provides them with a broader range of experiences and networking opportunities to engage in impactful translational outcomes such as commercialization and licensing with companies.

As BME has developed, so too have the variety and differentiation of specializations at each of its frontiers, resulting in a convergence of disparate technologies and expertise that cuts across disciplinary boundaries. Convergence as a discipline can be defined as “the coming together of insights and approaches from originally distinct fields” [26], for our discipline integrating knowledge and tools from the life and health sciences and physical, mathematical, and computational and engineering sciences. By merging diverse areas of expertise through partnership, convergence stimulates innovation, from basic science discovery to translation, for tackling scientific and societal challenges that exist at the interfaces of multiple fields [27]. The need for convergence in BME grows from the challenges that society faces to address the current limitations in medicine and the life sciences [28]. New frameworks and paradigms are needed, including simulating patient-specific models in silico, biomanufacturing of patient-specific tissues ex vivo, and innovating new biotechnology that can safely treat and cure diseases in vivo at the fundamental genetic level. Convergence in BME 2.0 provides curriculum that blends the boundaries of traditional disciplines by bringing together interdisciplinary researchers. As part of the curriculum, biomedical engineers need formal training on engineering design and leadership dynamics in interdisciplinary teams in rapidly evolving, constrained environments.

**Fig. 4.**
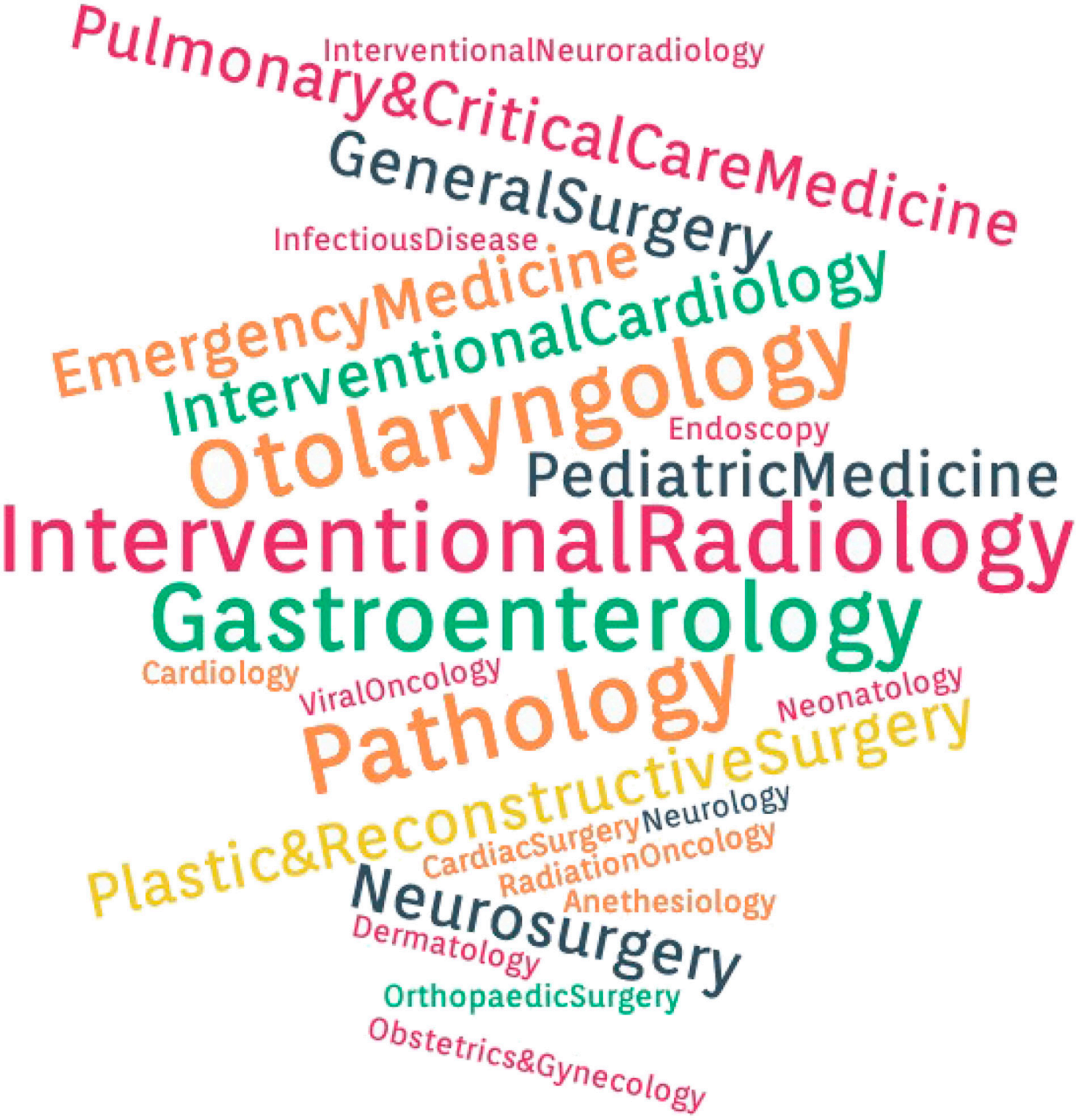
Preponderance word cloud of departments associated to 2019 to 2020 biomedical engineering project teams at Johns Hopkins University.

BME as a relatively young field is already embracing new fields of science and new “convergent technologies” that appear disparate but are incorporated in fundamental ways. The “unsiloed” approaches of convergence lead to new solutions to problems at the intersections of the pharamaceutical, medtech, and digital healthcare industries. As an example, at Johns Hopkins, the BME students are building the bridges for the convergence of new technologies for applications to projects in human health between the schools of medicine and engineering. Figure 4 shows a frequency word cloud depicting the clinical department preponderance for the BME design team projects at Johns Hopkins from 2019 to 2020, illustrating the broad range of interdisciplinary clinical applications that are impacting the future of healthcare.

An example of a curriculum exemplifying convergence is one launched at Johns Hopkins in biomedical data science called precision care medicine, a class bringing together BME and Anesthesia and Critical Care Medicine using de-identified patient data in the education and training of undergraduate and graduate students. The projects are defined by the clinical departments of the school of medicine with teams of 5 to 6 students, the emphasis being on creating teams with a diversity of students including undergraduate and graduate students, medical and/or nursing students, clinical residents and fellows, and clinical faculty. Datasets involve publicly available clinical datasets from the Medical Information Mart for Intensive Care database and the Philips eICU database [29], as well as datasets collected by John Hopkins Medical Institute (JHMI) physicians as part of institutional review board (IRB)-approved clinical studies that conform to the institutional policies regarding human subjects research. Specifically, students in this course take online training courses explaining the use of protected health information, as well as use of these datasets consistent with the IRB protocols governing their collection. Policies regarding use of JHMI patient data in this class were established by the JHMI Data Trust, a committee whose members work to assure appropriate and secure use of patient data.

### Champion an educational culture of inclusive excellence

Diversity in BME is critical to success in education, research, translation, and impact to society. Diversity is a key component of excellence in all branches of science bringing new ideas and expanding creativity—a key driver of discovery, design, and entrepreneurship. Including diverse perspectives will make our design teams, research laboratories, and start-ups more impactful to society. In fact, as argued by the National Academies, bringing dimensions of diversity to the maintenance of the academic program is inclusive excellence.

As defined by the National Academies [30]:

Inclusive excellence is a philosophical approach to higher education administration and processes that means attending to both the demographic diversity of faculty/students and the need for developing climates and cultures in institutions so that all have a chance to succeed in STEMM (Science, Technology, Engineering, Mathematics, and Medicine).

Clearly, diversity and convergence of ideas present the opportunity for more powerful innovation and greater excellence. Bringing diverse voices together in a successful BME program is both desirable and is already ongoing in many BME departments today [31]. BME 2.0 recognizes and affirms this critical component of BME in the 21st century. Championing and progressing diversity in BME 2.0 requires our commitment to equity beginning with recruitment of diverse populations. Faculty recruitment from underrepresented groups is an important catalyst for building inclusive environments and eliminating bias. We view efforts to engage recruitment and retention at all scales as essential. Programs such as that which have emerged from Columbia and JHU honoring and promoting Rising Stars [32] represent positive action that will greatly impact the cultural diversity of our discipline.

Thinking about specific practices, we envision curriculum changes that collectively can move our BME community toward a culture that is more inclusive and more equitable. Specific programmatic practices that we envision are to introduce into the curriculum classes that explore equitable outcomes in healthcare and BME’s role in attaining that. Our universities are rolling out new design projects and curriculum that engages the students in examining BME’s role in bringing biomedical solutions to our communities. Johns Hopkins has introduced the Health Inequity Design Challenge [33], asking first-year students to work in project design teams to solve a problem in health inequity in society as defined by the World Health Organization.

BME departments can also play a role in instilling in our colleagues and trainees the leadership qualities that embody inclusivity and equitable action. For those departments that participate in the Accreditation Board for Engineering and Technology (ABET) process, the program education objectives (PEOs) provide us with the opportunity to define broad program goals that we expect our trainees to attain subsequent to their graduation. Our Universities are defining PEOs that advocate for equitable access to the field and the technologies it produces by advancing diversity, inclusivity, and accessibility in the profession [34]. Finally, we can incentivize our faculty to participate by introducing into our departmental faculty evaluations criteria of merit which value activities that build a more diverse, equitable, and inclusive culture.

At the same time as ending systemic biases it is necessary to truly welcome students of all identities into BME. We recognize that the goal is not assimilation into one BME monoculture but rather to enable students from all groups and backgrounds to feel welcome and at home in BME and empowered to speak out about their own individual experiences and perspectives. Specifically, the recommendations here are that diversity and inclusion efforts are transformed by identifying intentional actions, requiring a shift in priorities and a shift in policy. This we believe is exemplified by the new Council of Diversity whose mission is to bring together representatives from every department to discuss and amplify successful practices for all types of departmental structures and available resources.

### Codify into the curriculum ongoing discoveries at the frontiers of the discipline, thus ensuring BME 2.0 as a launchpad for training the future leaders of the biotechnology marketplaces

As argued by Linsenmeir at the Council of Chairs [35], empirical statistics as of 2004 demonstrate a strong historical core common to many of the 60 universities reporting course data, 40 of which were accredited at the time of the original Gatchell and Linsenmeir paper [17]. As taken from the 2004 report [17], approximately 50% or more of the BME departments had required courses within their departments that were (a) physiology, (b) mechanics, (c) statistics, (d) instrumentation, (e) transport phenomena, and (f) signal analysis. Similar distributions still exist today across >100 accredited undergraduate BME programs as presented by Linsenmeir [35] and summarized at the Fourth BME Education Summit at Case Western Reserve University in May 2019 [36].

Historically, the cores and requirements for the departments have been well aligned; however, Gatchell and Linsenmeir’s data emphasizes the dichotomy pointed out by Citron; BME has been historically the application of traditional engineering with physiology. While historic agreement among universities has been compelling, the future focus areas and core curriculum presumably represents the future of BME 2.0, a discipline focused on biomedical imaging and medical devices, biomechanics and mechanobiology, computational medicine, genomics and systems biology, digital health and biomedical data science, neuroengineering, and immuno, regenerative, and tissue engineering. While systems physiology has been a foundational class, now at all biomedical institutions we see an equal role for physiology at the molecular scales as part of the burgeoning fields of modern molecular biology. At the other end of the quantitative spectrum, while circuits and statistics have been a mainstay for the medical technology industries, modern engineering programs are developing data science and machine learning curriculum at a pace that should be paralleled for BME.

In thinking about defining the next-generation core curriculum, we take inspiration from physics organized from the smallest quantum scales to the classical macro scales. This is natural for BME organizing the core around the microscales of molecules and cells to the macroscales of the systems physiology of organs and tissues. In physics, the core covers the abstractions associated to the fundamental forces starting from Newton’s laws; for building system biology models including both linear and nonlinear cell networks, linear and nonlinear signal analysis and control theory is arguably the proper abstraction. For BME, the core should be defined through its intellectual support of the focus areas.

The accompanying Table shows the natural transition of the BME 2.0 core demonstrating the push–pull interplay between focus areas and the core. The core tracks the marketplace because the advanced focus area curriculum track the industry markets including biotechnology and pharmaceuticals (https://medtech.pharmaintelligence.informa.com/), and medical technology industries (https://medicalalley.org/2017-year-in-review/) which are continuing to grow, develop, and pivot depending on advances and limitations of new technology.

**Table.** Original BME 1.0 core courses and transition to BME 2.0 core courses.

BME 1.0	BME 2.0
Physiology	Molecular biology and physiology
Organic chemistry	Organic chemistry (pre-med)
Programming	Python and Matlab
Statistics	Statistics and biomedical data science
Circuits	Signals, systems, controls
Transport	Systems biology
Thermodynamics	Statistical physics
Materials	Biomaterials/molecular engineering

The focus area classes should reflect a curriculum that supports and prepares students for the marketplace, and thus, we recommend developing a sequence of classes with names that are well recognized by the respective industries in which BME students are engaged.

We also see the continued rollout of BME 2.0 as having a strong influence on ABET and ABET requirements. As BME is a convergence of biology, medicine, and engineering, several of the BME 2.0 courses are neither just science or just engineering, but instead an integration of the two. This highlights the discipline of BME in the 21st century and how it is different from the more traditional engineering disciplines. Statistical physics is thermodynamics, but with the focus on the microscales of molecules inside of cells, instead of the macroscale. Students receive additional thermodynamics training within the biochemistry and molecular engineering course as well. These courses integrate both science and engineering would count fractionally, 50%/50% or 67%/33% in their engineering credit tally. Systems biology is a quantitative engineering course, not a pure science course, and can also count as engineering. As shown in Fig. 2, the BME 2.0 curriculum also includes focus area and upper-level elective courses where all students take additional engineering course work in a focus area for engineering depth. Some of these may be in the more traditional BME sequences that align with a focus area (e.g. transport and thermodynamics in the case of tissue and regenerative engineering), while other electives apply to other focus areas such as data science, machine learning, or genomic engineering. BME 2.0 programs would ensure that each student will graduate with the associated ABET engineering credits successfully satisfied.

Another principle of BME 2.0 is that each school features its own strengths, with the advanced curriculum reflecting those strengths. The focus areas can be personalized to the institution building on their individual program strengths and differentiators incorporating the elements of BME 2.0 that capitalize on their unique strengths. The University of Connecticut and UConn Health is leveraging its strengths in regenerative engineering [37], bringing convergence technologies for materials and stem cell science, developmental biology, and clinical translation for the regeneration of complex tissues launching the first master's degree and predoctoral training programs in regenerative engineering funded by an NIH T32 grant [38]. At Johns Hopkins, strengths within pathology, immunology, and BME have resulted in the creation of a new NIH-supported Translational ImmunoEngineering National Center for Biomedical Imaging and Bioengineering (NCBIB) as have Columbia’s strengths establishing the long-running Tissue Engineering Resource Center, with University of Maryland, Rice, and Wake Forest establishing a Center for Engineering Complex Tissues [39]. Similarly, the University of Pittsburgh leverages strengths associated to its proximity to the University of Pittsburgh Medical Center, and the Centers for Biotechnology and Bioengineering and Medical Innovation, with tracks in cellular engineering and medical product engineering.

We see flexibility in the matching of the curriculum to the local environment. At the same time, the value proposition that biomedical engineers offer in the biomedical science arena is that we are quantitative. Biomedical data sciences is uniquely an area that all BME programs could develop, with data wrangling a part of the core value of biomedical engineers. This is playing out in several of the universities. The University of Virginia’s (UVA) thrust with its new school of data science positions it well for biomedical data sciences with the school’s first dean—a former associate director of Data Science for the NIH and now a professor of BME. Virginia’s core curriculum reflects these strengths including the fact that students can high-throughput sequence their own DNA, with the 2 BME data science courses at UVA focused on genomics, bioinformatics, and gene expression. At Purdue, the Weldon School of BME approved a new data science requirement for all BME students recognizing that the successful integration of engineered medical technologies with digital healthcare delivery modes for diagnosis and treatment is becoming central to translation in personalized medicine. At Weldon, the requirements for core and elective courses in data science for all BME graduates began in 2018. In 2020, a new core course in data science and data analytics for all BME students was implemented. UMN BME has launched over the past 3 years a digital health subplan that has been crafted in partnership with the Department of Computer Science, representing an intentional effort to design courses to best prepare students for the myriad of emerging positions for managing and mining big data.

**Fig. 5.**
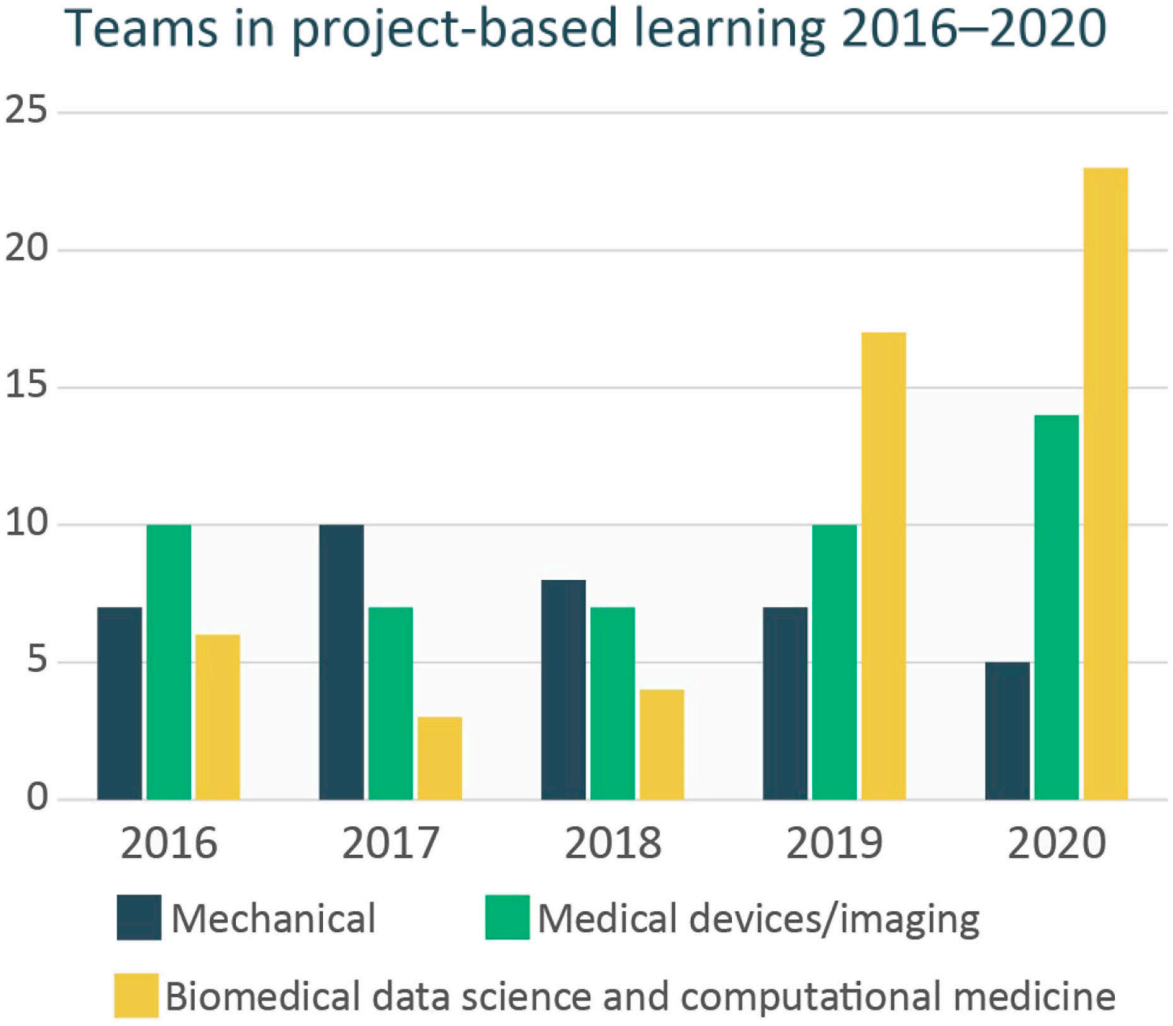
Project-based learning teams in the areas of mechanical devices, medical devices/imaging, and biomedical data science and computational medicine showing a rapid growth in data science projects since the inception of the 2017 core curriculum in biomedical data science at Johns Hopkins. Mechanical refers to projects where the solution uses mechanical actions, surgical tools, catheter systems, percutaneous feeding tubes, etc.

Johns Hopkins is seeing a rapid growth in biomedical data science interest in their BME student bodies. Examining the design teams enrollments, we see a strong proliferation of biomedical data science projects. Figure 5 depicts the growth of data science and computational projects at Johns Hopkins within the project-based learning parts of the program.

## BME Industry Marketplace Trends

We conclude our perspective by finishing on what we believe is a particularly high note for the 21st century of biomedical and biological engineering. While the industries focused around BME may have historically been relatively nascent, we see strong markets in biotechnology, pharmaceuticals, and medical technologies. BME 2.0 and the future leaders that emerge from it will play a major role in transforming the local economies. Klepper [40] argues this more generally—that the wealth of regions is greatly affected by the university ecosystem within it, and that universities play major roles in changing the economic ecosystem of their locales.

As one example, we take Purdue University, which is now centrally located in a rich ecosystem of orthopedic, medical device, and pharmaceutical industries and partners broadly within the life science industry. Purdue BME has acted as a key driver of economic development within the state of Indiana. The value of a BS/MS degree in BME for the local economy is clear. Purdue's Weldon School of BME reports Purdue graduates average 60% placement into industry positions directly with a BS or BS/MS Weldon School. Purdue has launched more than 30 Indiana medical device and life science companies, raising more than $100 million in venture funding and licensing more than 50 patented technologies to Indiana medical device and pharmaceutical companies. In total, more than 6 million patients worldwide have been directly helped by medical products based on these licensed technologies.

**Fig. 6.**
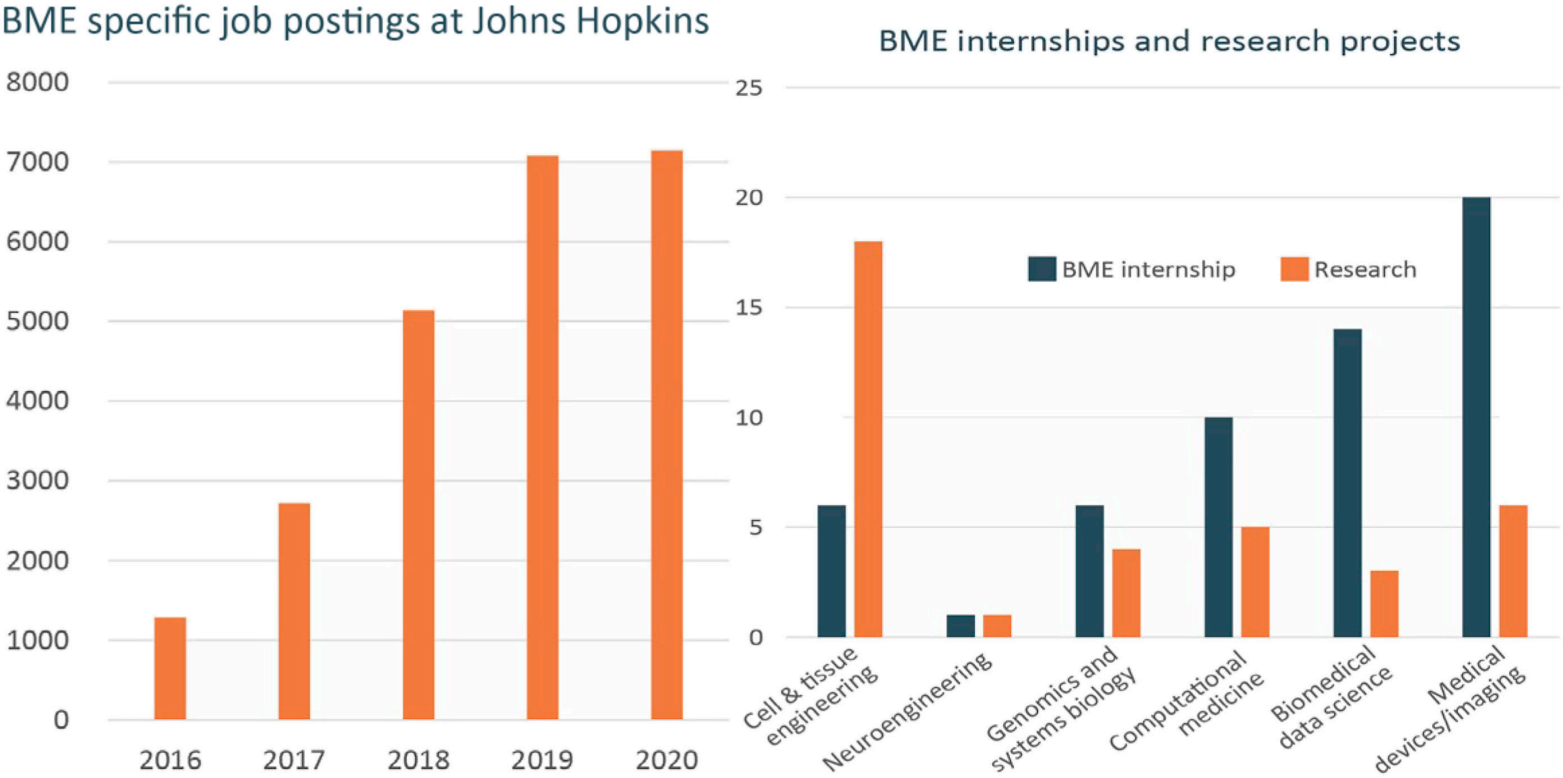
Left: Growth of total number of BME postings in Johns Hopkins online jobs. Right: Summer internship for Johns Hopkins Department of Biomedical Engineering juniors showing growth in data science, computation, and imaging.

With the emergence of biomedical science with engineering at its heart, we should expect to see continued transformation of the economy facilitated by the innovation that is emerging from our BME 2.0 programs. Historically, while interest from industry in hiring BME BS graduates may have been less apparent, this is no longer true for the biomedical science discovery industries. For example, at Johns Hopkins, there is rapid growth in industry interest in BME students as depicted in Fig. 6 (left), showing recent data on a dramatic increase in BME job postings. Similarly, we see significant participation of BME students in industry internships, especially from the biomedical data science, computational, and medical technology focus areas as depicted in Fig. 6 (right).

**Fig. 7.**
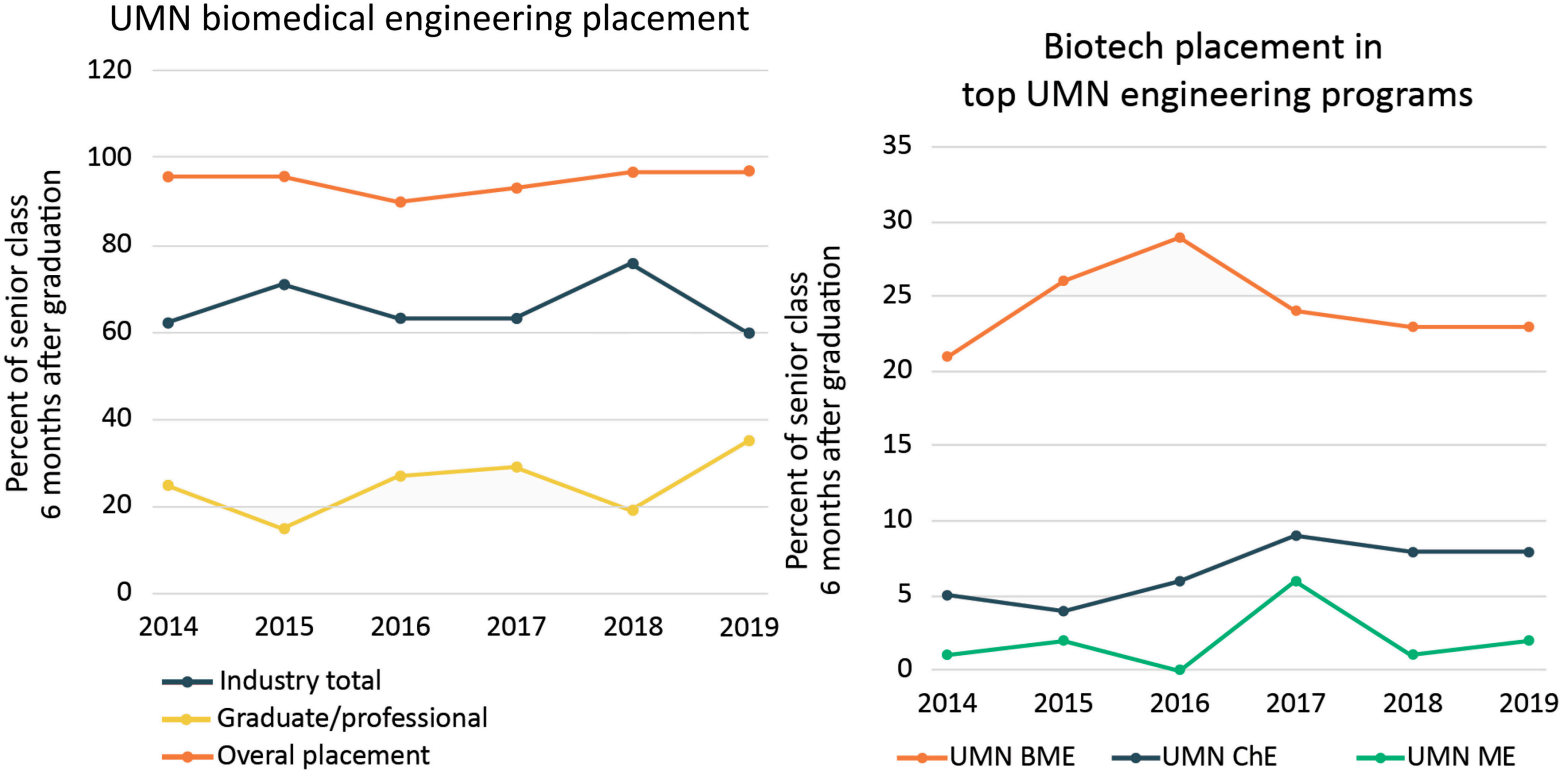
Left: Placement of BME graduates in industry far exceeds other career paths in regions with emphasis on medical technology. Right: Placement of BME graduates in biotech industry exceeds traditional engineering disciplines. Data taken from Department of Biomedical Engineering at University of Minnesota.

In fact, the notion that the BME industry may prefer hiring students from other disciplines such as EE or ME to students graduating from BME departments also does not appear to be true, with clear evidence that the first-tier medical technology companies prefer biomedical engineers for recruitment. The UMN proximity to the medical technology industry as part of the Twin-Cities area provides a university ecosystem that is integrated with medical device technology. Engagement with industry faculty occurs through curriculum such as computer-aided design and computer-aided engineering and prototyping, with the capstone projects mentored by leaders from local companies and faculty working together. The close interplay between industry and the curriculum translates into approximately 70% of UMN BME undergraduates employed by a diverse industrial landscape compared to approximately 40% on average in BME departments across the country [41] (Fig. 7, left). In addition, industrial partners prefer BME graduates over ME or ChE graduates from UMN (Fig. 7, right).

We close on a note that was stressed at 2019 Biomedical Engineering Education Summit as part of the academic core competencies and industry-ready skills themes. Industry's goal to hire BMEs with strong engineering skills appears to align precisely with BME 2.0 in which we have argued for more in-depth study and advanced differentiated curriculum focused on the engineering disciplines. This is enabled by taking BME courses from day 1, coupled to basic and advanced programming and data analytics capabilities as evidenced by the emphasis on BME incorporating biomedical data science as a core discipline in the curriculum.

## Conclusion

Since its inception more than half a decade ago, the discipline of BME has had profound scientific and societal impact. Arguably, its most significant impact has been to change the very nature of biological research and practice of medicine. It is now common for life scientists to harness engineering approaches in their research, and increasingly life scientists are being trained at the undergraduate and graduate levels in similar ways to biomedical engineers. As biomedical technology breakthroughs permeate the marketplace and biomedical research continues to become more quantitative and data intensive and dependent on novel technologies, we are poised to redefine the discipline once again and to change the way we train biomedical engineers for tomorrow. This transition emphasizes the importance of foundational learning of biology and engineering in the first 2 years of undergraduate education, with biology and engineering being taught in the context of each other. The junior and senior years emphasize both building depth in the specialized areas of BME that have emerged over the past several decades, and putting knowledge into practice through project-based learning. Convergence and inclusive excellence are emphasized throughout all years of training. Students pursue the key approaches of working as a team and learn self-motivation through the personalized design of their own curriculum.

We emphasize that the BME 2.0 perspective is designed from the point of view of the famous quote from Wayne Gretzky on “where the puck is going to be.” The process of transition from BME 1.0 to BME 2.0 is a process, one that will happen in different ways and on different time scales depending on the institution, its faculty, and the needs of its students. For example, even as increased attention in both medicine and industry is focusing on discovery and innovation on the molecular scale, training in physiology will remain an important aspect. To what extent physiology remains a separate biology course versus becoming integrated into the curriculum of “systems” BME courses will depend on each program. BME programs that grew historically out of founding faculty trained in ChE may more quickly embrace aspects of the molecular focus, just as those BME programs founded by those trained in EE may more quickly embrace the centrality of biomedical data science for all BME students. Thus, BME 2.0 is presented here as a framework and roadmap for BME programs to consider as they navigate the curriculum changes necessary for the 21st century biomedical age. The principles of BME 2.0 will empower us to engineer the future of medicine and healthcare delivery for years to come.
