# Erratum to “BME 2.0: Engineering the Future of Medicine”

**DOI:** 10.34133/bmef.0028

**Published:** 2023-09-20

**Authors:** Michael I. Miller, Andrew O. Brightman, Frederick H. Epstein, K. Jane Grande-Allen, Jordan J. Green, Eileen Haase, Cato T. Laurencin, Elizabeth Logsdon, Feilim Mac Gabhann, Brenda Ogle, Chun Wang, George R. Wodicka, Raimond L. Winslow

**Affiliations:** ^1^ Department of Biomedical Engineering, Johns Hopkins University, Baltimore, MD, USA.; ^2^ Weldon School of Biomedical Engineering, Purdue University, West Lafayette, IN, USA.; ^3^ Biomedical Engineering, University of Virginia, Charlottesville, VA, USA.; ^4^ Department of Bioengineering, Rice University, Houston, TX, USA.; ^5^ Department of Biomolecular Engineering and Department of Orthopaedic Surgery, University of Connecticutt, Storrs, CT, USA.; ^6^ Department of Biomedical Engineering, University of Minnesota-Twin Cities, Minneapolis, MN, USA.

In the article “BME 2.0: Engineering the Future of Medicine” [1], the competing interests statement was inadvertently omitted by the publisher from the published version of the article. This has now been corrected in the PDF and HTML (full text).
